# Saliva as an Alternative Matrix for Pharmacokinetic Research and Therapeutic Drug Monitoring of the Antituberculosis Drug Pyrazinamide

**DOI:** 10.3390/antibiotics15020163

**Published:** 2026-02-03

**Authors:** Arnold J. Ndaro, Hadija H. Semvua, Charles M. Mtabho, Claudia A. W. Heijens, Lindsey H. M. Te Brake, Gibson S. Kibiki, Rob E. Aarnoutse

**Affiliations:** 1 Kilimanjaro Clinical Research Institute, Kilimanjaro Christian Medical Centre, Moshi P.O. Box 2236, Tanzania; h.semvua@kcri.ac.tz (H.H.S.); c.mtabho@kcri.ac.tz (C.M.M.); g.kibiki@kcri.ac.tz (G.S.K.); 2 Department of Pharmacy, Pharmacology and Toxicology, Radboud Institute for Medical Innovation, Radboud University Medical Center, 6525 GA Nijmegen, The Netherlands; lindsey.tebrake@radboudumc.nl (L.H.M.T.B.); rob.aarnoutse@radboudumc.nl (R.E.A.); 3 School of Pharmacy, KCMC University, Moshi P.O. Box 2240, Tanzania; 4 Regional Commissioner’s Office, Lindi P.O. Box 94, Tanzania; 5 Department of Clinical Pharmacy, Rijnstate Hospital, 6815 AD Arnhem, The Netherlands; cheijens@rijnstate.nl

**Keywords:** pyrazinamide, saliva, matrix, pharmacokinetics, therapeutic drug monitoring

## Abstract

**Introduction**: Plasma is the standard biological fluid used in pharmacokinetic (PK) studies and therapeutic drug monitoring (TDM) of pyrazinamide, a key antituberculosis (TB) drug. This study described the PK of pyrazinamide in saliva and investigated whether saliva could serve as an alternative matrix for pyrazinamide PK evaluations. **Methods**: Fifteen adult Tanzanian TB patients in the intensive treatment phase participated in a descriptive PK study. Time-matched saliva (stimulated using a Salivette^®^ with citric acid) and plasma samples were collected at multiple intervals up to 24 h after drug intake. Pyrazinamide concentrations were measured using validated HPLC methods, exposure measures were assessed, and predictive performance for salivary concentrations was determined. **Results**: Salivary exposure to pyrazinamide (AUC_0–24h_: 230 h·mg/L; C_max_: 28.6 mg/L) was lower than plasma exposure (AUC_0–24h_: 377 h·mg/L; C_max_: 36.4 mg/L, *p* < 0.001), but T_max_ was similar (median 2.0 h, *p* = 0.893). A saliva/plasma ratio of 0.59 was assessed, and a reciprocal conversion factor of 1.68 allowed for reasonably accurate (bias 5.8%) but imprecise (imprecision 24.3%) plasma concentration predictions from saliva. Use of a conversion factor of 1.49, based on more stable saliva/plasma concentration ratios for samples between 2 and 6 h post-dose, resulted in a bias of 0.74% and imprecision of 17.7% for predicting plasma concentrations from salivary concentrations in the 2–6 h interval. **Conclusions**: The exposure to pyrazinamide in saliva is relatively high. Salivary measurement of pyrazinamide can be used as a semi-quantitative predictor of pyrazinamide plasma concentrations.

## 1. Introduction

Tuberculosis (TB) remains a major global public health problem. In 2023, about 10.8 million people were diagnosed with active TB and 1.25 million patients reportedly died due to this infectious disease globally [1].

Pyrazinamide is a pivotal drug used in first-line TB therapy. It is a potent sterilizing agent that has been proven to help reduce the duration of TB treatment from 9 to 6 months [2,3]. Low exposure to pyrazinamide has been linked to poor treatment outcomes for TB [4,5], and the microbiological efficacy of pyrazinamide was found to increase with higher drug concentrations [6]. Further exposure-response evaluations are needed to identify the pyrazinamide dose that optimizes efficacy at acceptable safety and to assess its potential role in new TB treatment combinations. Other pharmacokinetic evaluations of pyrazinamide take place in the context of patient care, where plasma concentrations of pyrazinamide are measured and interpreted to individualize the dose of patients (Therapeutic Drug Monitoring, TDM) [7,8]. For TDM of pyrazinamide, samples are taken at 2 h and 6 h after the dose to assess (‘catch’) peak concentrations (C_max_) [8]. Alternatively, samples are taken at 2 h, 4 h and 6 h post-dose to estimate C_max_ [7] and also the total exposure to the drug (area under the concentration versus time curve, AUC_0–24h_) with a so-called limited sampling formula [9]. Samples can also be taken at other time points when a population pharmacokinetic approach (maximum a posteriori Bayesian fitting) is used (with appropriate software) to assess AUC_0–24h_ [10]. The reference range for pyrazinamide C_max_ values is 20–60 mg/L [8] and target values for AUC_0–24h_ are 363 h·mg/L [5] or 380 h·mg/L [9].

Plasma has been the main biological sample for pharmacokinetic studies and TDM of many drugs. The total (protein-bound plus unbound) drug concentration is most commonly measured, given the complexities associated with measuring unbound concentrations, which are able to penetrate tissues and exert biological activity. Salivary sampling may serve as an attractive alternative to blood sampling for pharmacokinetic studies and TDM [11,12,13]. It has been stated that the collection of saliva samples is simple and non-invasive, does not require the expertise of drawing blood, is associated with less stress, fear and discomfort especially in children, and avoids complications of infection and thrombosis. In addition, salivary concentrations may reflect protein-unbound concentrations [11,12,13].

Various studies have demonstrated the utility and challenges of using saliva as an alternative matrix for pharmacokinetic studies and TDM of the first-line TB drugs rifampicin and isoniazid [10]; however, so far, no such study has been performed for pyrazinamide.

The current study, therefore, aimed (1) to describe the pharmacokinetics of pyrazinamide in saliva and (2) to assess whether saliva could be an alternative matrix for pharmacokinetic studies and TDM of pyrazinamide.

## 2. Results

### 2.1. Patients

Fifteen Tanzanian TB patients were included in the study. Twelve of the patients (80%) were men. Their median age was 37 years (range 19–50 years), and their median body weight was 49.5 kg (range 41.5–73.6 kg).

### 2.2. Pharmacokinetics of Pyrazinamide in Saliva

**Figure 1:**
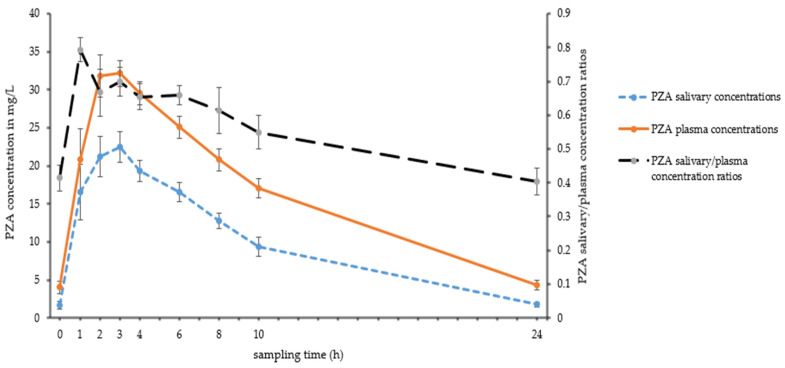
Salivary and plasma concentrations of pyrazinamide. Abbreviations: PZA: pyrazinamide. Depicted are geometric means of salivary and plasma concentrations of pyrazinamide at individual time points (Y axis, left) and geometric means of saliva/plasma concentration ratios (Y axis, right). Error bars represent standard errors of the mean. Each geometric mean and error bar value relates to 15 time-matched samples.

Intensive pharmacokinetic sampling for saliva and plasma occurred between days 18 to 21 of TB treatment. The geometric mean salivary AUC_0–24h_ and C_max_ of pyrazinamide were 230 h·mg/L and 28.6 mg/L, respectively, which were lower compared to those obtained in plasma (377 h·mg/L and 36.4 mg/L, respectively, Table 1). The between-patient variability in AUC_0–24h_ and C_max_ of pyrazinamide in plasma and saliva was limited (threefold or less, see Table 1). The T_max_ values in saliva and plasma were similar, with a median T_max_ of 2.0 h. Pyrazinamide was eliminated more quickly from saliva (t_1/2_ 5.8 h) compared to plasma (t_1/2_ 7.2 h, *p* = 0.002). Figure 1 shows the pharmacokinetic curves of pyrazinamide in saliva and plasma.

### 2.3. Evaluation of Saliva as Alternative Sampling Matrix for Pyrazinamide: Individual Concentrations Between 0 and 24 h Post-Dose

The expected number of individual saliva and plasma concentrations was as follows: 15 patients × 9 sampling points/patient = 135 concentrations. One pair of saliva and plasma pyrazinamide concentrations was not taken, and a total of 134 pairs of time-matched pyrazinamide concentrations in saliva and plasma were obtained. There was variation in saliva/plasma ratios over the dosing interval (Figure 1), which was confirmed by repeated-measures ANOVA, with ratios for the extreme time points (pre-dose, 1 h, 10 h and 24 h post-dose) being significantly different from others. Starting from T = 1 h post-dose, a significant linear trend to a decrease in the saliva/plasma ratio was observed (*p* = 0.001). The geometric mean saliva/plasma ratio for 134 ratios was 0.594 and the reciprocal conversion factor for prediction of individual plasma pyrazinamide concentrations from salivary concentrations was 1.684.

**Figure 2:**
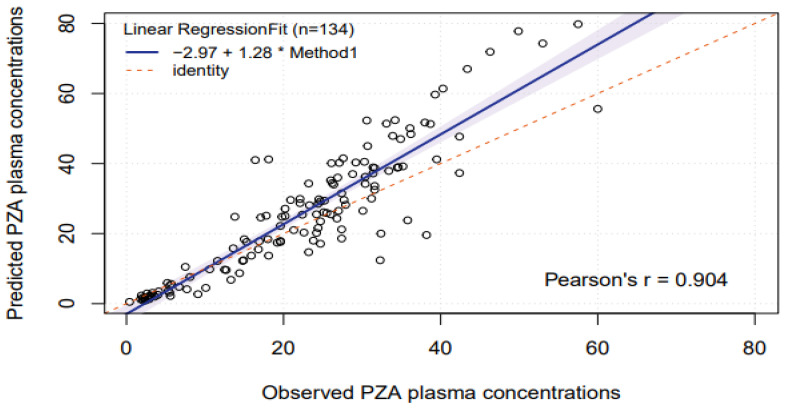
Linear regression of predicted and observed pyrazinamide plasma concentrations (0–24 h post-dose). Abbreviation: PZA: pyrazinamide. The figure shows a least squares linear regression analysis. Prediction of pyrazinamide plasma concentrations was based on measurement of salivary concentrations of pyrazinamide. The CI area is shaded. The intercept is −2.97 mg/L (95%CI −5.7 to −0.29 mg/L), and the slope is 1.28 (95%CI 1.18–1.39).

**Figure 3:**
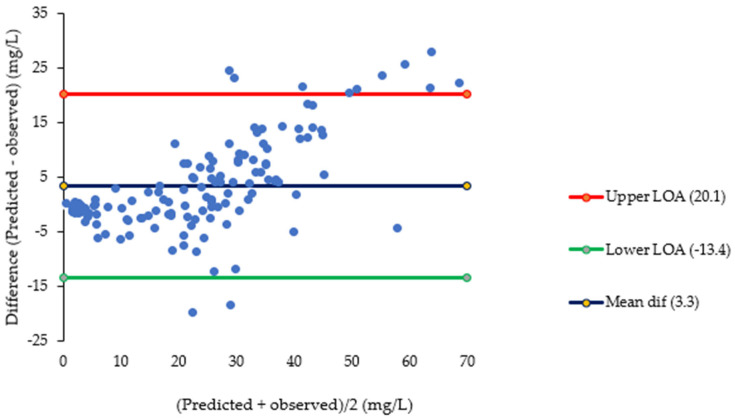

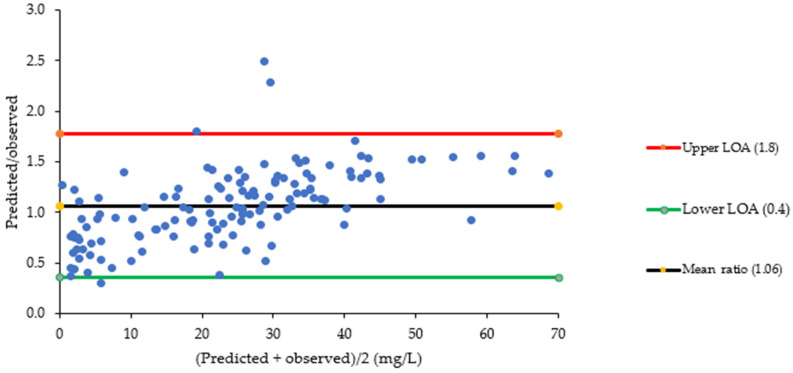
Bland–Altman plots of predicted and observed pyrazinamide plasma concentrations (0–24 h post-dose). Abbreviations: PZA: pyrazinamide, LOA: limit of agreement. The figure shows Bland–Altman plots. The upper plot shows the mean of the predicted and observed pyrazinamide concentrations (X axis) versus the difference between predicted and observed pyrazinamide concentrations (Y axis) in individual time-matched samples (n = 134) taken over the whole 24 h dosing interval. The lower plot shows the mean of predicted and observed pyrazinamide concentrations (X axis) versus the ratio of predicted/observed pyrazinamide concentrations (Y axis) in individual time-matched samples (n = 134) taken over the whole 24 h dosing interval.

After prediction of pyrazinamide plasma concentrations based on saliva concentrations, linear regression of predicted versus observed plasma concentrations showed a significant systematic bias of −2.97 mg/L and a significant proportional error of 28% (Figure 2). Bland–Altman plots (Figure 3) showed that the mean difference between predicted and observed pyrazinamide plasma concentrations was 3.3 mg/L and the mean ratio of predicted/observed pyrazinamide plasma concentrations was 1.06, with a wide range between the lower and upper limits of agreement (0.4–1.8). The median percentage prediction error (MPPE), obtained after the estimation of individual pyrazinamide plasma concentrations based on salivary concentrations, was 5.8%, and the median absolute percentage prediction error (MAPE) was 24.3%; the latter was above the maximum 15–20% value that was set as the acceptance criterion.

### 2.4. Evaluation of Saliva as Alternative Sampling Matrix for Pyrazinamide: Individual Concentrations Between 2 and 6 h Post-Dose

**Figure 4:**
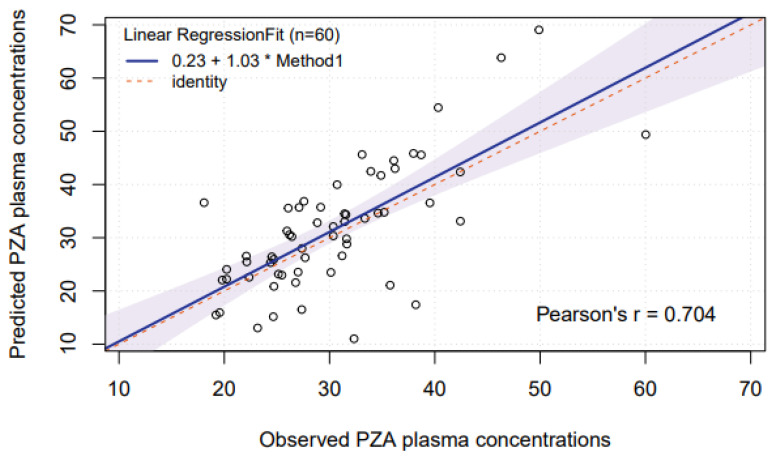
Linear regression of predicted and observed pyrazinamide plasma concentrations (2–6 h post-dose). Abbreviation: PZA: pyrazinamide. The figure shows a least squares linear regression analysis. Prediction of pyrazinamide plasma concentrations was based on measurement of salivary concentrations of pyrazinamide. The CI area is shaded. The intercept is 0.23 mg/L (95%CI −8.3 to 8.8 mg/L) and the slope is 1.03 (95%CI 0.76–1.30).

**Figure 5:**
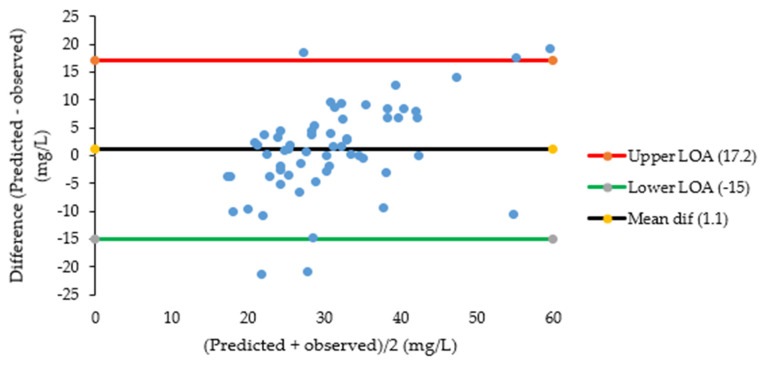

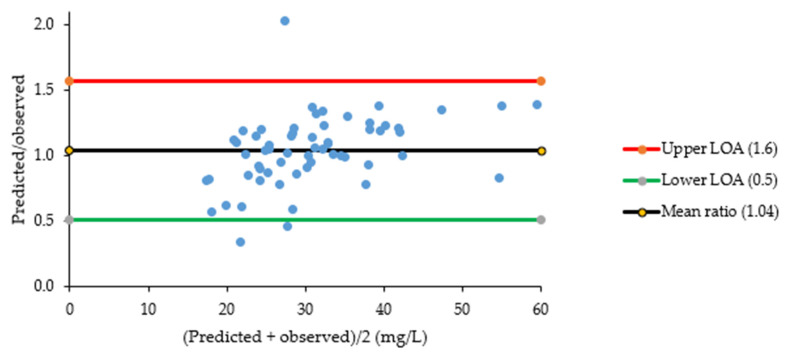
Bland–Altman plots of predicted and observed pyrazinamide plasma concentrations (2–6 h post-dose). Abbreviations: PZA: pyrazinamide, LOA: limit of agreement. The figure shows Bland–Altman plots. The upper plot shows the mean of the predicted and observed pyrazinamide concentrations (X axis) versus the difference between predicted and observed pyrazinamide concentrations (Y axis) in individual time-matched samples (n = 60) taken over the interval of 2–6 h post-dose. The lower plot shows the mean of predicted and observed pyrazinamide concentrations (X axis) versus the ratio of predicted/observed pyrazinamide concentrations (Y axis) in individual time-matched samples (n = 60) taken over the interval of 2–6 post-dose.

Based on the findings on samples taken between 0 and 24 h post-dose, an additional (non pre-planned) analysis was performed on samples taken between 2 and 6 h post-dose, the typical sampling window for TDM that showed a more stable saliva/plasma concentration ratio (Figure 1). When prediction of pyrazinamide plasma concentrations based on saliva concentrations was restricted to this 2–6 h time window, a conversion factor of 1.494 was assessed. In this case, linear regression showed no large or significant systematic or proportional bias (Figure 4). Blant–Altman plots are shown in Figure 5, displaying a somewhat less but still wide range between the lower and upper limits of agreement (0.5–1.6). In this case, MPPE was 0.74% and MAPE was lower and acceptable with 17.7%.

### 2.5. Evaluation of Saliva as Alternative Sampling Matrix for Pyrazinamide: AUC_0–24h_

The geometric mean AUC_0–24h_ saliva/plasma ratio was 0.610 (Table 1), and the reciprocal AUC_0–24h_ conversion factor was 1.639. The geometric mean AUC_0–24h_ saliva/plasma ratio for datasets of 14 patients ranged from 0.593 to 0.624 and the reciprocal AUC_0–24h_ conversion factor ranged from 1.603 to 1.690. In view of the limited number of participants and AUC_0–24h_ values, separate conversion factors for 14 patients were used to predict the plasma AUC_0–24h_ value in each 15th patient.

After such prediction of pyrazinamide plasma AUC_0–24h_ values based on salivary AUC_0–24h_ values, linear regression analysis showed no significant systematic or proportional bias (Supplementary Figure S1), but confidence intervals around the regression line, slope and intercept were broad. Blant–Altman plots showed a wide interval between the lower and upper limits of agreement, i.e., between 0.61 and 1.44 for the predicted/observed plasma AUC_0–24h_ of pyrazinamide (Supplementary Figure S2). Evaluation of predictive performance showed that the MPPE for estimation of pyrazinamide plasma AUC_0–24h_ values based on salivary AUC_0–24h_ values was 3.5% and MAPE was 16.7%, with the latter corresponding to the maximum 15–20% value that was set as acceptance criterion.

## 3. Discussion

The current study is the first to describe the pharmacokinetics of pyrazinamide in saliva and to assess whether saliva could be an alternative matrix for pharmacokinetic studies and TDM of pyrazinamide.

The first objective of this study was to describe the pharmacokinetics of pyrazinamide in saliva. Pyrazinamide appears to distribute rapidly into saliva. The exposure to pyrazinamide in saliva (AUC_0–24h_) can be considered relatively high, but it is lower than the exposure to total (protein bound plus unbound) pyrazinamide in plasma (Table 1, Figure 1). Most drugs enter saliva by simple diffusion from the bloodstream. Drug transport from blood to saliva therefore depends on physicochemical drug properties (molecular weight, lipid solubility, degree of ionization, and protein binding) and also on physiological factors such as salivary pH and salivary flow [14,15]. Pyrazinamide has a low molecular weight (123 g/mol) and is a very weak base (pKa = 0.5). At a salivary pH of 3 (as obtained with a Salivette^®^ [16]), it will be unionized. When unstimulated saliva is sampled (without the use of a Salivette^®^), a pH 7.5 is expected [16], and pyrazinamide will also be unionized. The protein binding of pyrazinamide in plasma is 1% (range 1–7%) [17]. Only free, non-protein-bound, unionized drug can pass from plasma to saliva. The physicochemical properties of pyrazinamide and its low protein binding (and therefore high free fraction in plasma) explain the relatively high pyrazinamide exposure in saliva. Although physiological factors such as salivary pH and saliva flow rate were not determined in our study, we expect these factors to have minor influence as we used the saliva stimulation sampling method with Salivettes^®^. This method is known to enable the production of a larger saliva sample volume in a shorter time, with a reduced pH gradient between plasma and saliva. It is also associated with decreased variability in saliva-to-plasma concentration ratios for certain drugs and results in fewer samples that are too viscous or discolored for drug analysis [18].

In our study, we observed variation in pyrazinamide saliva/plasma ratios in relation to time after the dose of pyrazinamide, with higher ratios in the absorption phase (Figure 1), despite the standardized sampling with the saliva stimulation sampling method. Such a decrease in saliva/plasma ratios has been described for the TB drugs isoniazid, rifampicin and moxifloxacin as well [10]. This variation is likely the result of arterial–venous differences in exposure to pyrazinamide. The concentration in saliva is in equilibrium with that in arterial blood, whereas the plasma concentration is determined in venous blood. During the absorption phase, the concentration of drug in arterial blood (reflected in saliva) is higher than that in measured venous blood [14,15]. After the absorption phase, the concentration ratio is expected to decrease. Whereas arterial–venous differences in exposure to pyrazinamide provide a plausible explanation for a decrease in pyrazinamide saliva/plasma ratios early after dosing, this hypothesis does not explain the continuing decrease in the ratio later in the dosing interval. This continuing decrease may be explained by somewhat faster elimination of pyrazinamide from saliva than from plasma (Table 1), but there may be other ongoing processes as well.

The second objective of this study was to assess whether saliva could be an alternative matrix for pharmacokinetic studies and TDM of pyrazinamide. Blant–Altman plots showed that 95% of the ratios of predicted/observed pyrazinamide plasma concentrations lied between 0.4 and 1.8, which is a wide range. This was also reflected in the evaluation of the performance of individual salivary pyrazinamide concentrations to predict plasma concentrations, which showed adequate bias (MPPE of 5.8%) but insufficient precision (MAPE of 24.3%). The use of a conversion factor based on a more stable saliva/plasma concentration ratio between 2 and 6 h post-dose narrowed the limits of agreement a bit to 0.5–1.6, and it improved MAPE to 17.7%, which is at the maximum 15–20% value that was set as acceptance criterion. This 2–6 h sampling time window is commonly used in TDM of pyrazinamide and other first-line TB drugs to estimate AUC_0–24h_ and to ‘catch’ C_max_ values [7,8,9]. The findings of our study indicate that salivary measurement of pyrazinamide can only be used as a semi-quantitative predictor of pyrazinamide plasma concentrations, preferably with measurements to be performed between 2 and 6 h post-dose. The same seems to be the case for the prediction of pyrazinamide plasma AUC_0–24h_ based on intensive saliva sampling, which showed a bias and imprecision of 3.5% and 16.7%, respectively, in our exploratory analysis with 15 participants.

As for TDM, this practice is described in treatment standards for TB [19] and is being applied in an increasing number of countries [20]. Salivary TDM is proposed as a non-invasive, patient-friendly alternative to blood sampling, which has the potential to overcome some barriers with traditional TDM [11,12,13]. Unfortunately, a large interpatient variation in saliva/plasma concentration ratio was observed for the first-line TB drug isoniazid, whereas prediction for rifampicin seemed feasible [21]. However, a large between-study variability in saliva/plasma ratios was observed for rifampicin, with some studies finding a ratio of 0.1–0.2 and others a ratio of 0.6 [11]. No salivary pharmacokinetic studies have been performed for the majority of other (second-line) TB drugs. A disadvantage of salivary TDM for TB drugs (also applicable to TDM in plasma/serum) is the requirement to use costly and labor-intensive HPLC or LC-MS/MS analyses that are not widely available. The availability of immuno-assays for pyrazinamide and other first-line TB drugs, to be used in any clinical chemistry/pathology laboratory, would be a large step forwards for TDM of TB drugs. Meanwhile, a mobile UV spectrophotometer may provide a simple solution for analyzing pyrazinamide drug concentrations in saliva samples [22].

A strength of our study is the applied methodology, which complies with most recommendations made in the literature for salivary PK studies, including the evaluation of actual patients, who use a recommended dosing regimen, fixed food intake, rinsing of the mouth before sampling, the use of paired saliva and plasma samples taken during the recording of a full pharmacokinetic curve, the standardization or measurement of salivary flow and pH, and the use of appropriate methods for bio-analysis and data processing [11].

Our study also has a few limitations. First, a relatively limited number of participants was included in the study (n = 15), but on the other hand all participants yielded nine matched plasma and saliva samples, resulting in 134 matched samples. Still patients were all from Tanzania and the majority was male. The use of saliva (as compared to plasma or serum) introduces additional interpatient variability related to salivary flow rate and pH, which we tried to standardize by using a Salivette^®^; however, other patient-related factors such as hydration status, oral health, and concurrent medications may also affect drug concentrations in saliva. Our study probably did not capture all sources of variability. Larger studies in other populations are thus needed to confirm our findings on the pharmacokinetics of pyrazinamide in saliva and the suitability of saliva as a matrix for pharmacokinetic evaluation and TDM of pyrazinamide. A second limitation of this study is that we confined to measurement of total (protein-bound plus unbound) pyrazinamide concentrations in plasma and did not measure unbound pyrazinamide, which may be better predicted based on salivary concentrations. On the other hand, it may be argued that the protein binding of pyrazinamide in plasma is probably very low (1%, range 1–7% [17]) meaning that total and unbound pyrazinamide concentrations are almost similar.

## 4. Materials and Methods

### 4.1. Study Design and Population

This was a descriptive pharmacokinetic study performed in Moshi, Kilimanjaro, Tanzania. Adult TB patients who had been under first-line TB treatment for at least two weeks (considering the expected ‘steady state’ of pyrazinamide, i.e., stable drug concentrations, at that time) in the intensive phase of TB treatment were eligible for inclusion.

Patients with a body weight less than 50 kg used three fixed-dose combination (FDC) tablets per day (i.e., 225 mg isoniazid, 450 mg rifampicin, 1200 mg pyrazinamide and 825 mg ethambutol, FDC tablets from Sandoz, Mumbai, India), and patients with a body weight ≥ 50 kg used four FDC tablets per day (i.e., 300 mg isoniazid, 600 mg rifampicin, 1600 mg pyrazinamide and 1100 mg ethambutol). Intensive pharmacokinetic sampling for both plasma and saliva took place during 24 h.

This was an explorative study; therefore, no sample size calculation was performed. The study aimed to recruit 15 patients, providing for an expected number of approximately 135 time-matched salivary and plasma concentrations of pyrazinamide.

The study participants were recruited from the outpatient TB clinic at the Mawenzi Regional Referral Hospital, Moshi, Tanzania. Pharmacokinetic sampling was performed at the clinical trial unit of the Kilimanjaro Clinical Research Institute, Moshi, Tanzania.

The study was approved by the Research Ethics Committee at the Kilimanjaro Christian Medical Centre (KCMC) University and by the Tanzanian National Institute for Medical Research. Patients were enrolled based on their voluntary consent to participate in this study.

### 4.2. Pharmacokinetic Sampling

The patients were asked not to eat for at least six hours (i.e., overnight) before the start of pharmacokinetic sampling. On the sampling day, the patients took their drugs under the supervision of a nurse in the morning and then had a standardized breakfast within 30 min after drug intake. The standardized breakfast consisted of a cup (250 mL) of tea with milk and sugar and two pancakes. Serial venous blood samples were collected just before, and 1, 2, 3, 4, 6, 8, 10, and 24 h after TB drug intake. Blood samples were centrifuged at 2800 rpm for 10 min. Aliquoted plasma was stored at −80 °C immediately.

Saliva was collected immediately (i.e., within 2 min) after every blood sample collection as follows. The patients were asked to rinse their mouths with water. Then, a Salivette^®^ was provided, a plastic device containing a dental cotton roll impregnated with citric acid that stimulates the salivary flow (Sarstedt, Etten-Leur, The Netherlands). The patients were asked to chew on the cotton roll inside the Salivette^®^. After one minute, they were asked to put the cotton roll back in the insert of the Salivette^®^ centrifuge vessel. The centrifuge vessel was centrifuged at 3000 *g* for 5 min, which allowed saliva to be collected in the extended tip of the Salivette^®^ tube. The saliva was put in polypropylene tubes and stored at −80 °C. The total process until storage in the freezer took a maximum of 45 min after sampling.

Both the plasma and saliva samples were transported on dry ice to the Department of Pharmacy, Pharmacology and Toxicology at Radboudumc, The Netherlands, which took approximately 24 h. Upon arrival, it was verified whether the samples were still deep-frozen in dry ice. The samples were then stored at −80 °C and subsequently subjected to bio-analysis within 2 weeks.

### 4.3. Bio-Analysis of Samples

Total (protein-bound plus unbound) plasma concentrations of pyrazinamide were measured with a validated HPLC method, as described before [23]. For the analysis of pyrazinamide in saliva, liquid–liquid extraction followed by HPLC was used. First, 100 µL of saliva was mixed with 50 µL of internal standard (nicotinamide) solution and 1 mL of ethylacetate/butanol (7:3% *v*/*v*) and the mixture was centrifuged at 4500 rpm for 5 min. Second, 850 µL of the upper organic layer was mixed with 200 µL 30 mM phosphoric acid and again the mixture was centrifuged at 4500 rpm for 5 min. Last, 160 µL of the lower water layer was added to a vial with 50 µL 0.5 M ammonium acetate buffer with pH 8.2 and 50 µL of this mixture was injected into the HPLC system (degasser SCM 1000, pump P4000, autosampler AS3000 and detector UV2000, all from Thermo Fisher Scientific, Bleiswijk, The Netherlands). The analytical column was a Polaris 3u C18-A column (150 × 4.6 mm ID) protected by a ChromSep guard Pol 3 C18-A 10 × 3 mm column (both from Varian, Middelburg, The Netherlands). The mobile phase consisted of 50 mM phosphate buffer with pH 7.0 and methanol, run in different proportions and at different flow rates during 28 min. UV detection was set at 265 nm. Accuracy and within-day precision ranged from 98 to 103% and from 2.1 to 5.5%, respectively, dependent on the concentration which ranged from 0.6 (limit of quantitation) to 60 mg/L. Linearity of the calibration line was assessed with linear regression analysis of detector response versus concentration of pyrazinamide (*p* was <0.05) and the correlation coefficient r (as a measure of the strength of the relationship) was >0.9. Analysis of stability of the samples indicated that, at −80 °C, pyrazinamide in saliva is stable for at least 3 weeks and in plasma for more than a year. Three freeze–thaw cycles did not affect the stability of pyrazinamide in saliva or plasma.

### 4.4. Pharmacokinetic Data Analysis

The area under the plasma versus concentration time curve (AUC_0–24h_), the highest observed concentration (C_max_), time to maximum concentration (T_max_), and elimination half-life (t_1/2_) were assessed for pyrazinamide by using noncompartmental pharmacokinetic methods in WinNonLin Version 5.3 (Pharsight Corp., Mountain View, CA, USA), as described before [24].

### 4.5. Statistical Analysis: Description of the Pharmacokinetics of Pyrazinamide in Saliva

Saliva and plasma pharmacokinetic parameters AUC_0–24h_, C_max_, and t_1/2_ were presented descriptively (geometric mean, minimum, and maximum values). Median, minimum, and maximum were calculated for the categorical variable T_max_. Differences in paired pharmacokinetic parameters in salivary and plasma concentrations were assessed using paired *t*-tests on log-transformed pharmacokinetic parameters, while the Wilcoxon signed-rank test was used to assess differences in T_max_ in saliva and plasma.

### 4.6. Statistical Analysis: Saliva as Alternative Matrix for Plasma (Individual Concentrations)

First, ratios of individual saliva concentrations versus individual plasma concentrations of pyrazinamide were calculated for all time-matched samples. Next, the effect of sampling time on the pyrazinamide concentration ratios was assessed using repeated measures (within-subject) ANOVA on the log-transformed saliva/plasma concentration ratios. A geometric mean was calculated for all saliva/plasma concentration ratios. The reciprocal plasma/saliva concentration ratio was used as conversion factor to estimate pyrazinamide plasma concentrations based on the measured salivary concentrations.

The agreement between direct measurement of pyrazinamide plasma concentrations and predicted plasma concentrations based on saliva concentrations was assessed using (1) linear regression analysis, (2) an evaluation of Bland–Altman plots, and (3) a quantification of predictive performance. Regression analysis assesses the presence of a systematic bias (intercept ≠ 0) or a proportional error (slope ≠ 1). Ordinary least squares linear regression was chosen as type of regression. This type of regression is chosen when one method serves as a reference method or gold standard [25], which was the case for direct measurement of pyrazinamide in plasma with our assay that was extensively validated internally and that performed well in an external proficiency testing program [26]. Least squares regression assumes no error in the reference method results [25]. Bland–Altman plots show the bias between two methods, limits of agreement (LOA) that encompass 95% of differences, and the variability in agreement over the concentration range [27]. Finally, the predictive performance of estimations of individual pyrazinamide plasma concentrations based on saliva measurements was assessed by a jackknife analysis [28]. This means that calculation of conversion factors was based on datasets in which one out of the expected 135 samples was omitted, followed by use of these conversion factors to estimate the plasma concentrations of the 135th sample. Such estimations were performed for all 135 expected samples. A jackknife analysis is useful in cases of small sample size, such as this study, and is an alternative to using a so-called index patient group to derive a prediction formula and a second validation group to validate the formula [28]. Potential bias in the predictions was assessed using the median percentage prediction error (MPPE, [29]). This is the median of all percentage prediction errors, which were defined as follows: 100% × (predicted plasma concentration—measured plasma concentration)/measured plasma concentration. Imprecision was assessed using the median absolute percentage prediction error (MAPE). This is the median of all absolute percentage prediction errors. The absolute percentage prediction error was defined as follows: 100% × absolute ((predicted plasma concentration − measured plasma concentration)/measured plasma concentration). For acceptable predictive performance, the MPPE and the MAPE should not be more than 15–20% [28].

### 4.7. Statistical Analysis: Saliva as Alternative Matrix for Plasma (AUC_0–24h_ Values)

It was also evaluated whether pyrazinamide plasma AUC_0–24h_ values could be assessed using salivary AUC_0–24h_ values. In view of the limited number of participants and AUC_0–24h_ values recorded (n = 15), this analysis was regarded as exploratory. The AUC_0–24h_ values of pyrazinamide in saliva and plasma were evaluated in the same way as the matched individual samples. Thus, a saliva/plasma ratio for AUC_0–24h_ was calculated for each participant, a geometric mean saliva/plasma AUC_0–24h_ ratio was assessed, and a reciprocal conversion factor was used to estimate pyrazinamide plasma AUC_0–24h_ values based on the salivary AUC_0–24h_ values. Again, (1) linear regression analysis was performed and (2) Bland–Altman plots were evaluated to assess the agreement between the observed plasma AUC_0–24h_ values and those predicted based on saliva AUC_0–24h_ values. In this case, (3) predictive performance was assessed by calculating conversion factors based on datasets in which 1 out of the 15 patients was omitted, followed by use of these conversion factors to estimate the plasma concentrations of the 15th patient and calculation of MPPE and MAPE.

Statistical analyses were performed with IBM SPSS Statistics (version 31, IBM, Armonk, NY, USA), an interactive website (https://bahar.shinyapps.io/method_compare/ (accessed on the 23 December 2025) [25], for regression analyses), and Microsoft Excel (Redmond, WA, USA, for Blant Altman plots and predictive performance).

## 5. Conclusions

The exposure to pyrazinamide in saliva is relatively high, which can be explained by its physicochemical characteristics and limited protein binding in plasma. Individual pyrazinamide plasma concentrations and plasma AUC_0–24h_ values can be predicted from salivary concentrations with sufficient bias, but imprecision is large, meaning that salivary measurements provide a semi-quantitative estimate of pyrazinamide plasma concentrations at the most.

## Figures and Tables

**Table 1:** Pharmacokinetics of pyrazinamide in saliva and plasma (n = 15).

PK Parameter	Saliva	Plasma	*p* Values	Saliva/Plasma Ratios
AUC_0–24h_ (mg/L)	230 (139–421) [195–272]	377 (280–588) [327–435]	<0.001	0.61 (0.45–0.90) [0.55–0.68]
C_max_ (mg/L)	28.6 (19.3–47.4) [24.5–33.3]	36.4 (26.0–60.0) [31.9–41.5]	<0.001	0.79 (0.58–1.01) [0.72–0.86]
T_max_ (h)	2.03 (1.00–6.00)	2.03 (1.00–6.00)	0.893	–
t_1/2_ (h)	5.8 (4.4–9.0) [5.1–6.5]	7.2 (5.6–10.0) [6.5–8.0]	0.002	0.81 (0.52–1.16) [0.72–0.91]

Abbreviations: PK: pharmacokinetics, AUC_0–24h_: area under the time versus concentration curve, C_max_: maximum concentration, T_max:_ time to maximum concentration, t_1/2_: elimination half-life. All parameters are in geometric means and ranges (between brackets) and 95% confidence intervals (between square brackets), except for T_max_, which is in median and range. A paired *t*-test was applied to log-transformed pharmacokinetic parameters, except for T_max_, for which the Wilcoxon signed-rank test was used.

## Data Availability

Data of this study will be freely available at the Department of Pharmacy, Pharmacology and Toxicology, Radboudumc data repository and at the KCRI at a repository. Data requests should be through h.semvua@kcri.ac.tz and rob.aarnoutse@radboudumc.nl.

## Supplementary Materials

The following supporting information can be downloaded at https://www.mdpi.com/article/10.3390/antibiotics15020163/s1: Supplementary Figure S1: Linear regression of predicted and observed pyrazinamide plasma AUC_0–24h_ values; Supplementary Figure S2. Bland–Altman plots of predicted and observed pyrazinamide plasma AUC_0–24h_ values.
