# Supplementary material for: Saliva as an Alternative Matrix for Pharmacokinetic Research and Therapeutic Drug Monitoring of the Antituberculosis Drug Pyrazinamide
Source: Antibiotics (Basel). 2026 Feb 3;15(2):163. doi: 10.3390/antibiotics15020163 (PMC12937273; doi:10.3390/antibiotics15020163)
Supplement: Supplementary file 1 [file antibiotics-15-00163-s001.zip › Supplementary Figure S1.pdf]

Supplementary Figure S1. Linear regression of predicted and observed pyrazinamide plasma $AUC_{0\text{-}24h}$ values[1]

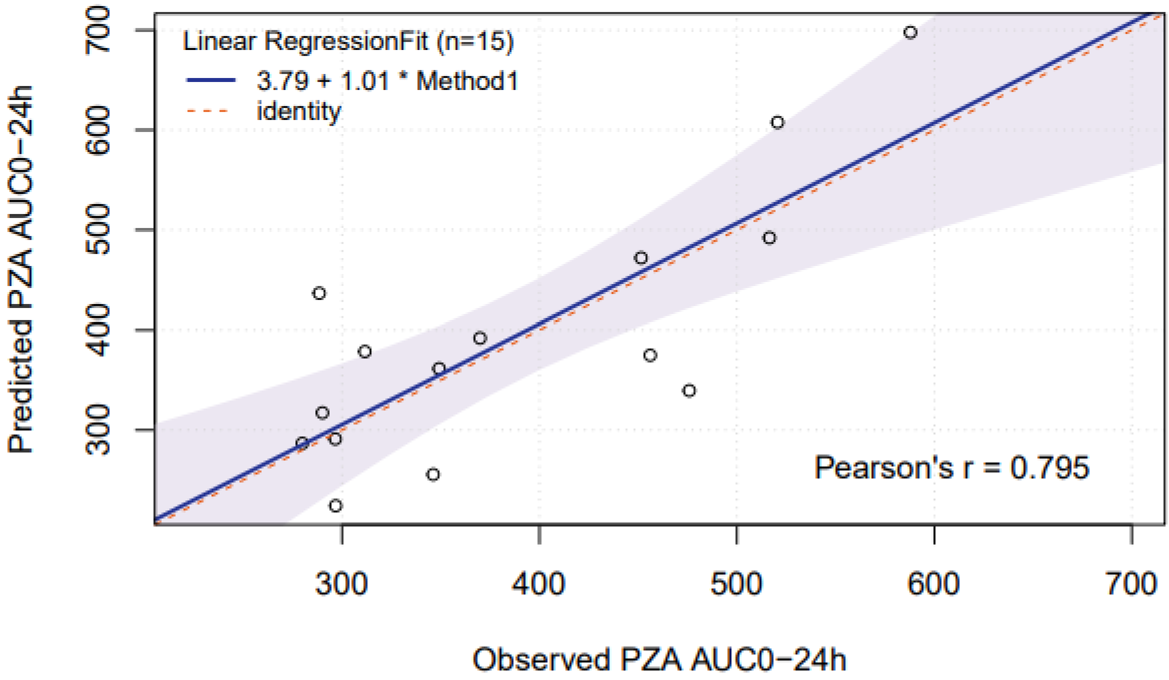

1. Abbreviation: PZA: pyrazinamide.
   The figure shows a least squares linear regression analysis. Prediction of pyrazinamide plasma $AUC_{0\text{-}24h}$ values was based on measurement of salivary $AUC_{0\text{-}24h}$ values of pyrazinamide. The CI area is shaded.
   The slope is 1.01 (95%CI 0.55-1.47) and the intercept is 3.8 mg/L (95%CI -181 to 188 mg/L)
