# Supplementary material for: Saliva as an Alternative Matrix for Pharmacokinetic Research and Therapeutic Drug Monitoring of the Antituberculosis Drug Pyrazinamide
Source: Antibiotics (Basel). 2026 Feb 3;15(2):163. doi: 10.3390/antibiotics15020163 (PMC12937273; doi:10.3390/antibiotics15020163)
Supplement: Supplementary file 1 [file antibiotics-15-00163-s001.zip › Supplementary Figure S2.pdf]

Supplementary Figure S2. Bland-Altman plots of predicted and observed pyrazinamide plasma AUC_{0-24h} values

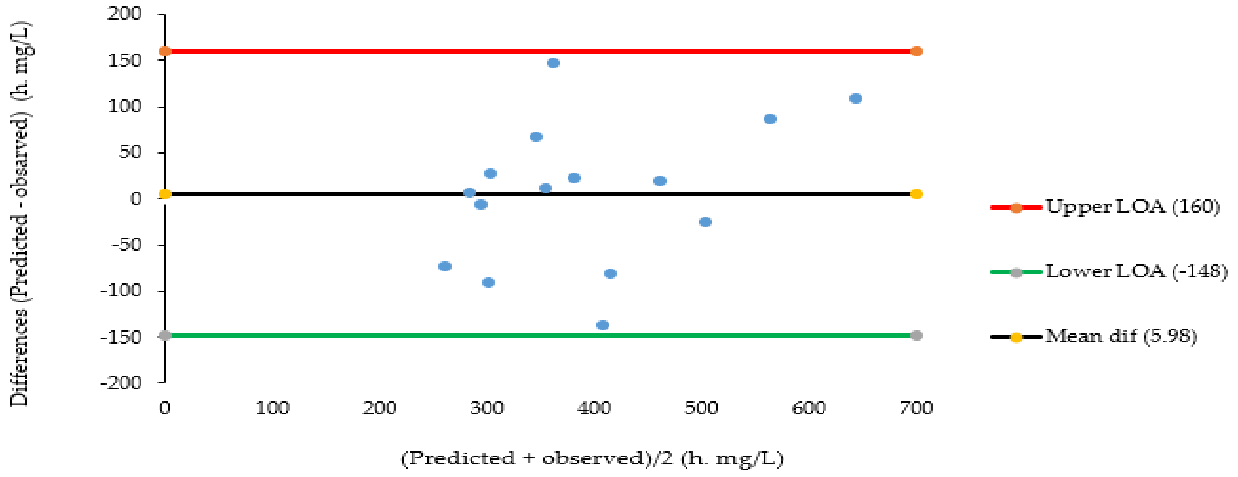

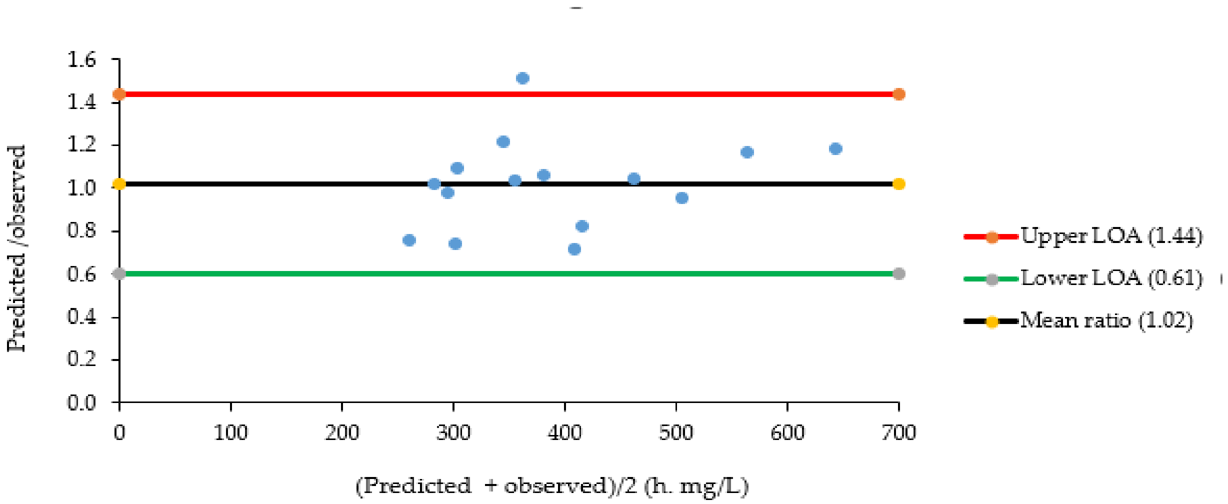

1. Abbreviations: PZA: pyrazinamide, LOA: limit of agreement
The figure shows Bland–Altman plots.
The upper plot shows the mean of the predicted and observed total exposure to pyrazinamide (AUC_{0-24h}, X axis) versus the difference between predicted and observed pyrazinamide AUC_{0-24h} values (Y axis) in patients (n=15).
The lower plot shows the mean of predicted and observed pyrazinamide concentrations (*X* axis) versus the ratio of predicted/observed pyrazinamide AUC_{0-24h} values (Y axis) in patients (n=15).
